# Microstructural Characteristics of the Weighted and Directed International Crop Trade Networks

**DOI:** 10.3390/e23101250

**Published:** 2021-09-26

**Authors:** Yin-Ting Zhang, Wei-Xing Zhou

**Affiliations:** 1 School of Business, East China University of Science and Technology, Shanghai 200237, China; ytzhang@mail.ecust.edu.cn; 2 School of Mathematics, East China University of Science and Technology, Shanghai 200237, China; 3 Research Center for Econophysics, East China University of Science and Technology, Shanghai 200237, China

**Keywords:** econophysics, international crop trade network, microstructural properties, weighted networks, directed networks, network metrics

## Abstract

With increasing global demand for food, international food trade is playing a critical role in balancing the food supply and demand across different regions. Here, using trade datasets of four crops that provide more than 50% of the calories consumed globally, we constructed four international crop trade networks (iCTNs). We observed the increasing globalization in the international crop trade and different trade patterns in different iCTNs. The distributions of node degrees deviate from power laws, and the distributions of link weights follow power laws. We also found that the in-degree is positively correlated with the out-degree, but negatively correlated with the clustering coefficient. This indicates that the numbers of trade partners affect the tendency of economies to form clusters. In addition, each iCTN exhibits a unique topology which is different from the whole food network studied by many researchers. Our analysis on the microstructural characteristics of different iCTNs provides highly valuable insights into distinctive features of specific crop trades and has potential implications for model construction and food security.

## 1. Introduction

Due to a steep increase in global population, the demand for food is increasing rapidly and may continue to do so for decades [1]. Getting rid of starvation and achieving food security are global key aims, as emphasized in the Sustainable Development Goals in the 2030 Agenda [2]. In various parts of the world, the local production of food cannot fulfill their internal demands [3]. International food trade has become a crucial element for feeding the world’s population [4]. It is obvious that only through international trade can the food supply of the global population be better secured [5]. Propelled by the development of transportation and technology for grain storage, the international food trade network (iFTN), covering most parts of the world, is playing an increasingly significant role [6]. Thus, we analyzed international trade networks for four important crops (maize, rice, soybean, and wheat), which are the main sources of calories for human consumption [7] and also serve as feed for livestock. Our aim was to understand the microstructural characteristics of these international crop trade networks (iCTNs). The descriptive analysis of network properties may have implications for food security.

Network analysis is a convenient tool for characterizing the international food trade system. The iFTN usually has a broad distribution with a fat tail, implying a heterogeneous network structure [8]. Network analysis has also been applied to quantify the structural characteristics of food networks, such as betweenness, centrality, and clusters to identify whether some economies form clusters or a certain economy is at the center of a large cluster [9]. The dynamics of the iFTN signify the increasing globalization of food trade through the evolution of node degree, node strength, link weight, and other topological properties [10]. Studies on such topics usually focus on the aggregate network of food trade [11,12] or one kind of goods, such as seafood [13,14,15], meat [16], or agro-food [12,17,18]. Over the past two decades, a number of researchers have studied the structure and evolution of iCTNs, including those of maize [19], wheat [3,6,20], and soybean [21]. However, most international crop trade studies focused only on one important crop or combined several crops into an aggregate network. Little research has compared different crops or explored the relationships between the topological properties of different iCTNs.

To fill this research gap, we attempted to analyze the microstructural characteristics of four iCTNs, differing from previous studies that each focused on an iCTN of a single crop or the aggregate iCTN of several crops. Using the standard datasets of international crop trade over the period from 1986 to 2018, we describe the degree distributions and weight distributions for different crops in different years. We then discuss the relationships among microstructural properties, including node degrees, node strengths, link weight, reciprocity, and clustering coefficients. We found that different international crop trade systems have unique trade patterns. Our analysis on the microstructure of different crop networks provides valuable insights into the global food system for the evaluation of food security.

The remainder of this paper is organized as follows. Section 2 describes the datasets used in our work and the construction of the international crop trade networks. Section 3 presents the empirical analysis and results of four iCTNs. We summarize conclusions in Section 4.

## 2. Datasets and Network Construction

### 2.1. Data Description

The datasets on international crop trade we analyzed were retrieved from the food trade matrix dataset of the Food and Agriculture Organization (FAO), http://www.fao.org, accessed on 15 September 2021. We selected four major crops (wheat, maize, rice, and soybean) which cover more than 50% of the global calorie intake. In particular, wheat ensures the 20%, rice 16%, maize 13%, and soybean 8% of the global human calorie intake [4]. In addition, soybean exports account for three quarters of the livestock feed worldwide [22]. Our datasets cover the period from 1986 to 2018 and 246 economies.

### 2.2. Network Construction

For each crop *c* in each year *t*, we constructed the network based on the international cross-border trade flows $w_{ij}^{c}\left(t\right)$, where nodes *i* and *j* represent two economies that are connected by different types of links, and *c* represents different crops. The entry in $w_{ij}^{c}\left(t\right)$ represents the amount of crop *c* in US dollars that flows from economy *i* to economy *j* in year *t*. Hence, $w_{ij}^{c}\left(t\right)$ is a time-dependent network in which the nodes correspond to economies involved in the international trade of crop *c*. In the rest of our work, V denotes the set of nodes in the network, $e_{ij}$ denotes the link from node *i* to node *j*, and E denotes the set of links in the network.

For each trade flow, there should be two records in the data, one for exporting economy *i* and the other for importing economy *j*. In our analysis, $w_{ij}^{c}\left(t\right)$ is defined as the import value of importing economy *j* from exporting economy *i*. If the import data are missing, we used the corresponding export value of exporting economy *i* to importing economy *j*. For simplicity of presentation, we drop the superscript *c* in the rest of this work, hoping it will not cause confusion.

**Figure 1:**
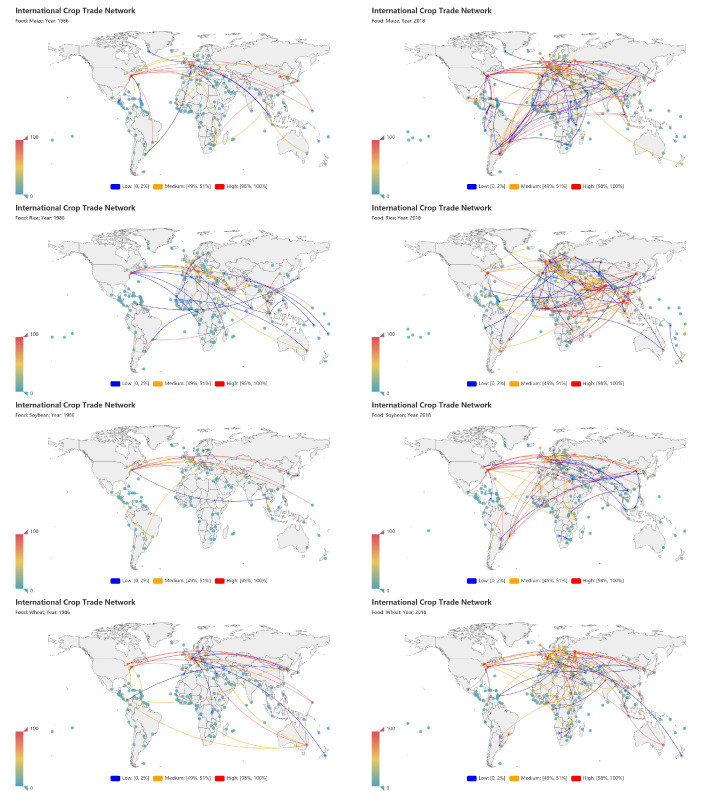
International crop trade networks (iCTNs) in 1986 (left column) and 2018 (right column). The rows from top to bottom, respectively, describe maize, rice, soybean, and wheat. For clarity, in each map we have shown only the links with high, medium, and low trade values, each accounting for 2% of the total number of links.

Figure 1 shows the iCTNs under investigation. The rows from top to bottom represent trade flows for maize, rice, soybean, and wheat, respectively, in 1986 and in 2018. To better illustrate results for each crop, only the links whose trade values ranked 98–100% (high percentage), 49–51% (medium percentage), and 0–2% (low percentage) are shown. We observed that there were more edges in 2018, indicating the network grew rapidly from 1986 to 2018 in terms of trade value. The structure of each network has significantly changed in the past 30 years.

## 3. Empirical Results

### 3.1. Node Degrees

The degree $k_{i}$ of a node *i* is defined as the number of nodes connected with node *i* in the network. For a directed network, node *i* has the in-degree $k_{i}^{\mathrm{in}}$ and the out-degree $k_{i}^{\mathrm{out}}$, respectively, measuring numbers of links flowing from and to other nodes. The in-degree $k_{i}^{\mathrm{in}}$ of node i∈V is defined as follows.

(1) $$k_{i}^{\mathrm{in}}=\sum_{j\in V-\{i\}}I_{E}\left(e_{ji}\right)=\sum_{j=1}^{N_{V}}I_{E}\left(e_{ji}\right),$$

where $I_{E}\left(e_{ji}\right)$ is the indicator function:

(2) $$I_{E}\left(e_{ji}\right)=\left\{\begin{array}{cc} 1, & \mathrm{if}\;\;e_{ji}\in E\\ 0, & \mathrm{if}\;\;e_{ji}\notin E \end{array}\right.$$

Similarly, the out-degree $k_{i}^{\mathrm{out}}$ of node i∈V is defined as follows.

(3) $$k_{i}^{\mathrm{out}}=\sum_{j\in V-\{i\}}I_{E}\left(e_{ij}\right)=\sum_{j=1}^{N_{V}}I_{E}\left(e_{ij}\right),$$

The total degree $k_{i}$ of the node *i* is thus

(4) $$k_{i}=k_{i}^{\mathrm{in}}+k_{i}^{\mathrm{out}}.$$

**Figure 2:**
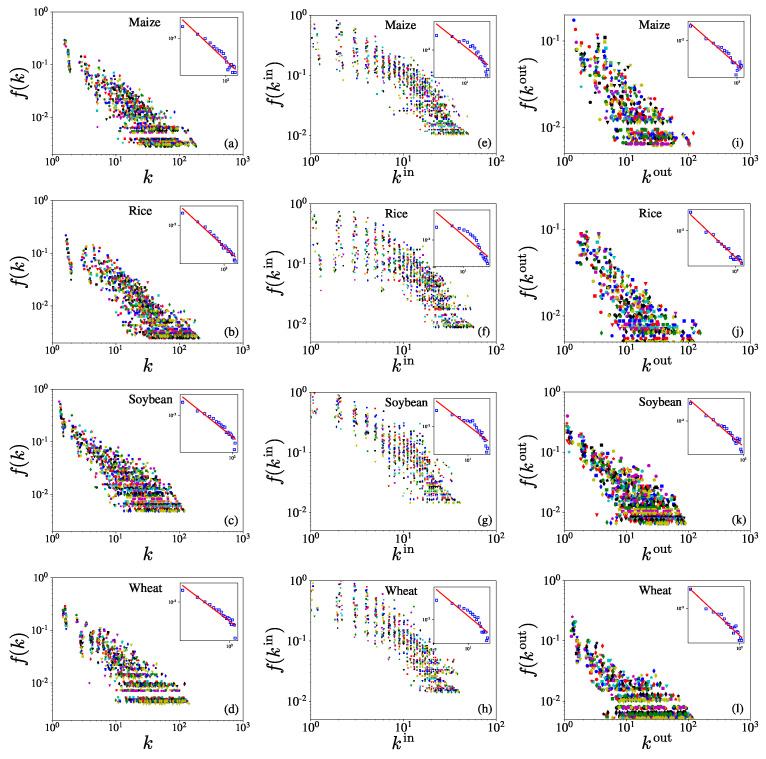
Empirical distributions of total degrees *k* (left column, **a**–**d**), in-degrees $k^{\mathrm{in}}$ (middle column, **e**–**h**), and out-degrees $k^{\mathrm{out}}$ (right column, **i**–**l**) for the four iCTNs over the period from 1986 to 2018. The rows from top to bottom show the distributions for maize, rice, soybean, and wheat. For each graph, the inset presents the distribution of all degrees over 33 years and the corresponding power-law fit. Different colors of the symbols correspond to different years. For each iCTN, the total degrees *k* and the out-degrees $k^{\mathrm{out}}$ follow power-law distributions. The $R^{2}$ values of the fitting distributions of the total degrees for maize, rice, soybean, and wheat are, respectively, 0.9161, 0.97036, 0.8904, and 0.8816. The $R^{2}$ values of the fitting distributions of the in-degrees are, respectively, 0.8911, 0.9655, 0.9309, and 0.9346. The $R^{2}$ values of the fitting distributions of the out-degrees are, respectively, 0.8911, 0.9655, 0.9309, and 0.9346.

Figure 2 illustrates the empirical distributions of total degrees *k* (left column), in-degrees $k^{\mathrm{in}}$ (middle column), and out-degrees $k^{\mathrm{out}}$ (right column) for the four iCTNs over the period from 1986 to 2018. The insets show the aggregate distributions on the log–log scale, together with the power-law fits. In each plot, we can observe that the distributions have similar shapes but with evident differences over the years. For degrees of each type in each row, the distributions also look similar to some extent. It was found that, for each iCTN, the total degrees *k* and the out-degrees $k^{\mathrm{out}}$ follow power-law distributions:

(5) $$f\left(k\right)∼k^{-\alpha -1},$$

where α=1.11 for the maize network, α=0.89 for the rice network, α=1.11 for the soybean network, and α=0.99 for the wheat network. The $R^{2}$ values of the fitting distributions of the total degrees for maize, rice, soybean, and wheat are, respectively, 0.9161, 0.97036, 0.8904, and 0.8816.

For the international trade network of all commodities, the total degree follows a power-law distribution [23]. For the international rare earths trade network, the total degree follows a power-law distribution in each year from 1986 to 2015 [24]. For the international cereal trade network, the total degree follows a power-law distribution in 1986 and an exponential distribution in 2013 [25].

Figure 2 also shows that the out-degree distribution has a power-law tail:

(6) $$f\left(k^{\mathrm{out}}\right)∼{\left(k^{\mathrm{out}}\right)}^{-\alpha^{\mathrm{out}}-1}$$

where $\alpha^{\mathrm{out}}=0.84$ for the maize network, $\alpha =1.03^{\mathrm{out}}$ for the rice network, $\alpha =0.81^{\mathrm{out}}$ for the soybean network, and $\alpha =0.60^{\mathrm{out}}$ for the wheat network. The $R^{2}$ values of fitting distribution of out-degree are, respectively, 0.8911, 0.9655, 0.9309, and 0.9346. In contrast, the in-degrees $k^{\mathrm{in}}$ show a strong deviation from power-law distributions in some commodity trade networks. Nevertheless, the fat-tailedness of all distributions indicates that there are economies that export to many other economies and economies that import crops from other economies. The in-degree and out-degree distributions have different forms in different networks. Power-law distributions are reported for both the out-degree and in-degree of the international trade network of every commodity [23], and the international agricultural greenhouse gas network through international trade [26]. For the international maize trade networks over 2000–2009, the distributions of the in-degree and the out-degree exhibit inverse exponential behavior [19]. The USA food flow network exhibits a normal distribution for the in-degree and the out-degree [8].

**Figure 3:**
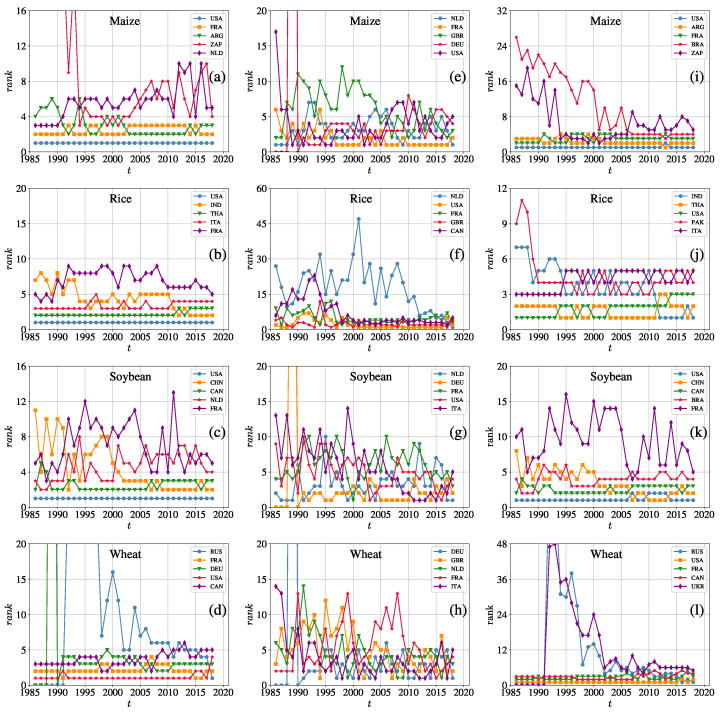
Top five economies’ rankings of total degrees *k* (left column, **a**–**d**), in-degrees $k^{\mathrm{in}}$ (middle column, **e**–**h**), and out-degrees $k^{\mathrm{out}}$ (right column, **i**–**l**) for the four international crop trade networks over the period from 1986 to 2018. For each iCTN, we selected economies that had the top largest total degrees, in-degrees, and out-degrees in 2018, and we show the evolution of their rankings from 1986 to 2018. The rows show the evolutionary rankings for maize, rice, soybean, and wheat from top to bottom. The ordinate represents the rankings of five economies in a certain year based on the values of the three indicators.

For each iCTN, we select the top five economies based on their total degrees, in-degrees, and out-degrees in 2018 and illustrate the evolution of rankings from 1986 to 2018 in Figure 3. It can be seen that the United States has the highest rankings of node degrees in the four networks, especially in the maize, rice, and soybean trade networks, and its total degree always ranks first (during 1986–2018) due to its high rankings of out-degree, but the rankings of its in-degree slightly fluctuate. This means that the USA has more export trade partners than import. The rankings of some economies’ degrees behave differently. After the collapse of the Soviet Union in 1991, Russia participated in international trade, and its number of trade partners gradually increased. It became the largest exporter of wheat worldwide in 2018, which is consistent with what the US Department of Agriculture reported in 2018. The department declared that Russia stayed ahead in wheat export trades. As for China, it is a large rice importer and it also imports soybeans and wheat. Overall, the rankings of node degrees in different iCTNs are distinct. In each network, the rankings of the total degrees and the out-degrees are steady, but the in-degree rankings fluctuate significantly.

**Figure 4:**
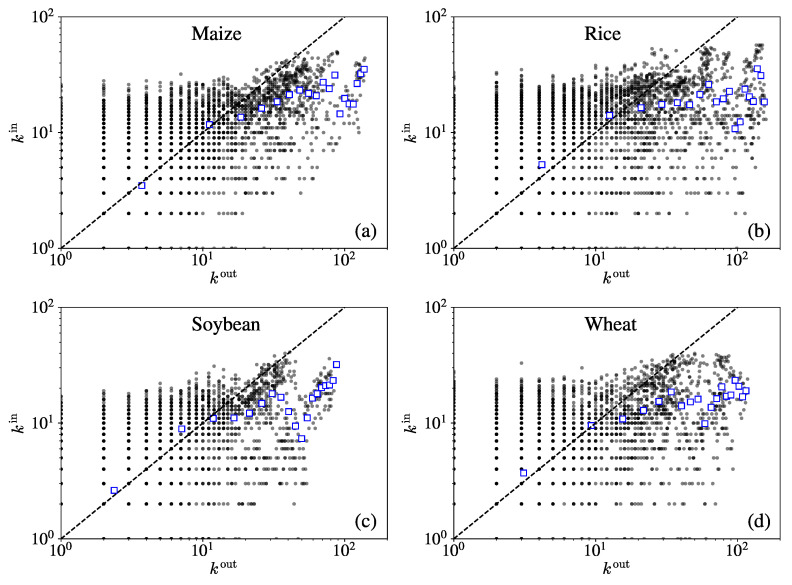
Relationship between the in-degree $k_{i}^{\mathrm{in}}$ and the out-degree $k_{i}^{\mathrm{out}}$ for the four crops –maize (**a**), rice (**b**), soybean (**c**), and wheat (**d**)—over the period from 1986 to 2018. The correlation coefficients between the in-degree $k_{i}^{\mathrm{in}}$ and the out-degree $k_{i}^{\mathrm{out}}$ for each crop network are, respectively, 0.6473, 0.4896, 0.6120, and 0.5341.

Figure 4 shows the relationship between the in-degree and the out-degree for the four iCTNs. It can be observed that scatter plots of the in-degree and the out-degree have similar patterns. There are more data points located below the diagonal. We can observe that there is no clear relationship between the in-degree and the out-degree, differing from the results reported for other goods, such as in the international plastic resin trade network [27].

### 3.2. Node Strengths

In weighted networks, the strength of a node is defined as the sum of link weights directly connected to the node. A given node has in-strength and out-strength in accordance with the trade flow. The in-strength of node i∈V represents the import value of economy *i* from other economies:

(7) $$s_{i}^{\mathrm{in}}=\sum_{j\in V-\{i\}}w_{ji}=\sum_{j=1}^{N_{V}}w_{ji},$$

where $w_{ji}$ means trade value from economy *j* to economy *i*. Similarly, the out-strength of node *i* is defined as follows:

(8) $$s_{i}^{\mathrm{out}}=\sum_{j\in V-\{i\}}w_{ij}=\sum_{i=1}^{N_{V}}w_{ij},$$

which is the export value of economy *i* to other economies.

**Figure 5:**
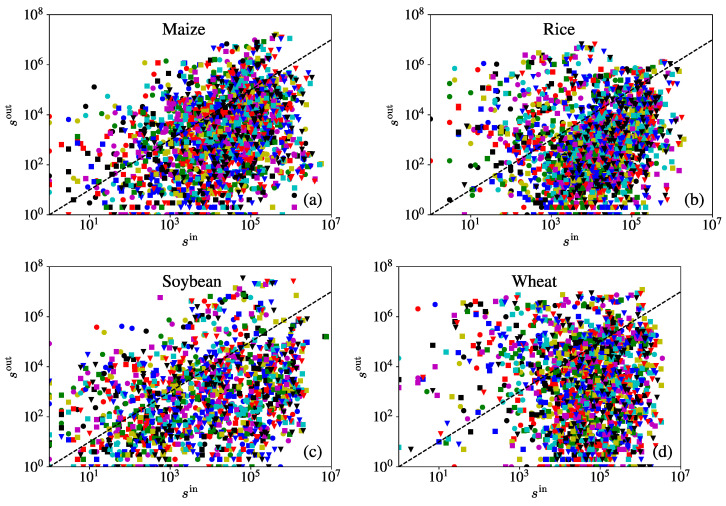
Relationships between the in-strength $s_{i}^{\mathrm{in}}$ and out-strength $s_{i}^{\mathrm{out}}$ for the four crops –maize (**a**), rice (**b**), soybean (**c**), and wheat (**d**)– over the period from 1986 to 2018. Different colors of the symbols correspond to different years. The in-strength and the out-strength are almost uncorrelated in each iCTN. The majority of data points are below the dashed line in each plot.

The relationships of the in-strength and the out-strength shown in Figure 5 indicate that they are almost uncorrelated (correlation coefficients are, respectively, 0.0569, 0.0581, 0.01830 and 0.0202), though Guo et al. have shown that the strength–strength curve is correlated with a positive slope around 1.0 in the world trade network for all commodities [28]. Moreover, we can see that the majority of data points are located below the dashed line in each plot, which means the in-strength is larger than the out-strength of most nodes. It indicates that most economies have a deficit in crop trade and need to import more than they export. A study on the wheat networks in 2009–2014 suggested that large exporters are in most cases also large importers, but the correlation between the in-strength and the out-strength is also very weak [3].

### 3.3. Degree vs. Strength

**Figure 6:**
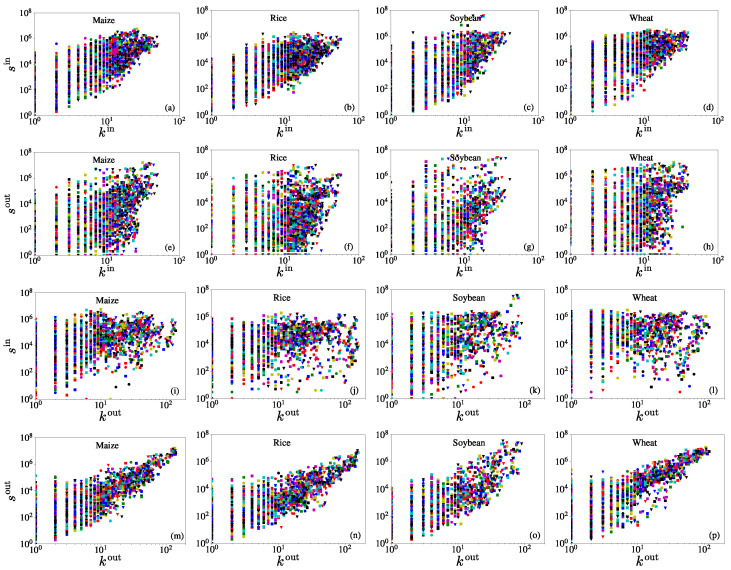
Relationships between strengths and degrees for the four iCTNs over the period from 1986 to 2018. From the top row (**a**–**d**) to the bottom row (**m**–**p**), the plots describe the relationships between $s_{i}^{\mathrm{in}}$ and $k_{i}^{\mathrm{in}}$, $s_{i}^{\mathrm{in}}$ and $k_{i}^{\mathrm{out}}$, $s_{i}^{\mathrm{out}}$ and $k_{i}^{\mathrm{in}}$, and $s_{i}^{\mathrm{out}}$ and $k_{i}^{\mathrm{out}}$. The columns from left to right, respectively, represent the international trade networks of maize, rice, soybean, and wheat. Different colors of the symbols correspond to different years. The in-strength is positively related to the in-degree and the out-strength is positively related to the out-degree. The correlation coefficients between the pairs of variables in (**a**–**p**), respectively, are 0.4056, 0.3664, 0.1666, 0.4722; 0.3048, 0.1368, 0.1653, 0.2725; 0.1940, 0.2385, 0.3574, 0.1566; and 0.6815, 0.6580, 0.4831, 0.7909.

The strength of a node is related to its degree, usually as a power law, for many socioeconomic networks [29,30]. In a trade network, an economy that has more trade partners will also have higher trade values [31]. In Figure 6, we show the relationships between strengths and degrees for the four iCTNs from 1986 to 2018. In all the cases, the in-strength increases with the in-degree (the correlation coefficients for each crop network are, respectively, 0.4056, 0.3664, 0.1666, and 0.4722) and the out-strength increases with the out-degree (the correlation coefficients for each crop network are, respectively, 0.6815, 0.6580, 0.4831, and 0.7909). This is consistent with the general perception that economies with more trade links tend to have large trade flows. We further observed that $s_{i}^{\mathrm{in}}$ is related to $k_{i}^{\mathrm{in}}$, and $s_{i}^{\mathrm{out}}$ is related to $k_{i}^{\mathrm{out}}$, both in an evident power-law form. In contrast, power-law relationships between $s_{i}^{\mathrm{in}}$ and $k_{i}^{\mathrm{out}}$ and between $s_{i}^{\mathrm{out}}$ and $k_{i}^{\mathrm{in}}$ are less evident. The power-law relationship has also been reported between $s^{\mathrm{out}}$ and $k^{\mathrm{out}}$ for the international trade networks of wheat and rice in two different periods (1992–1996 and 2005–2009) [20]. However, the relationships between strengths and degrees differ from the scale-free character of the USA-only food flows [8], and the international trade network of all commodities [32].

### 3.4. Link Weight

A link connecting two nodes is usually associated with weights that are vital to describing the connection traits of each link in a networked system. In a directed weighted trade network, how large the weight is represents how large the import or export trade flow is. A large number of studies have shown that weight distributions of many weighted networks are greatly heterogeneous [33].

**Figure 7:**
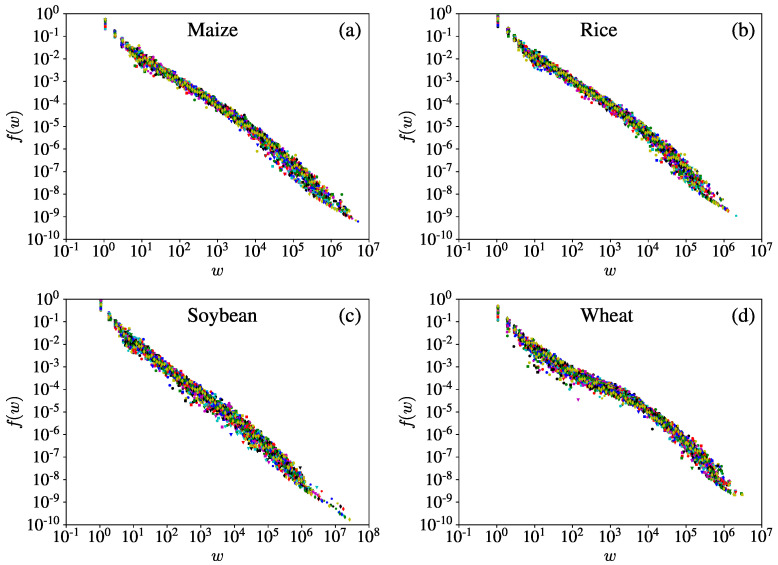
Empirical distributions of link weights for the four iCTNs: (**a**) maize, (**b**) rice, (**c**) soybean, and (**d**) wheat. In each plot, there are 33 yearly distributions corresponding to the 33 years from 1986 to 2018. Different colors of symbols correspond to different years. The link weight distributions of the maize, rice, and soybean trade networks exist in power-law form when the weights are not too large. The $R^{2}$ values of fitting distribution for maize, rice, soybean, and wheat trade networks are 0.7749, 0.7762, 0.8572, and 0.4815.

Figure 7 illustrates the yearly empirical distributions of link weights for the four iCTNs. For each crop, we present 33 distributions for the 33 yearly networks. For each crop, the distributions collapse onto a single curve with some deviations, implying that the formation of the international trade networks of a given crop in different periods is driven by common key mechanisms. Figure 7a–c show that the link weight distributions of the maize, rice, and soybean trade networks exist in power-law form when the weights are not too large, but decay toward the right tail. In contrast, the wheat networks in Figure 7d do not exhibit power-law scaling. It is obvious that the international wheat trade network differs from other iCTNs, implying that different mechanisms underlie the formation of different iCTNs. Thus, the analysis could lead to bias when studying the aggregated iCTN in terms of food security. In fact, the distributions of weights for maize, rice, and soybean are right skewed (most economies have relatively small weights), but for the wheat trade there were larger fractions of links with big export volumes in 1992 and 2018 [34].

### 3.5. Reciprocity

Link reciprocity plays an important role in shaping the directed networks and understanding the observed network topology [35]. A traditional definition of a node *i*’s reciprocity is the ratio of the number $k_{i}^{R}$ of reciprocal links of node *i* to the total number $k_{i}$ of links of node *i* [36]:

(9) $$R_{i}=\frac{♯\left(\left\{j:e_{ij}\in E\;\&\;e_{ji}\in E\right\}\right)}{♯\left(\left\{j:e_{ij}\in E\;\mathrm{or}\;e_{ji}\in E\right\}\right)}=\frac{k_{i}^{R}}{k_{i}},$$

where

(10) $$k_{i}^{R}=♯\left(\left\{j:e_{ij}\in E\;\&\;e_{ji}\in E\right\}\right)=\sum_{j\neq i}{\left(w_{ij}w_{ji}\right)}^{0}$$

is the number of reciprocal links node *i* has. In Equation (10), we pose $0^{0}=0$.

**Figure 8:**
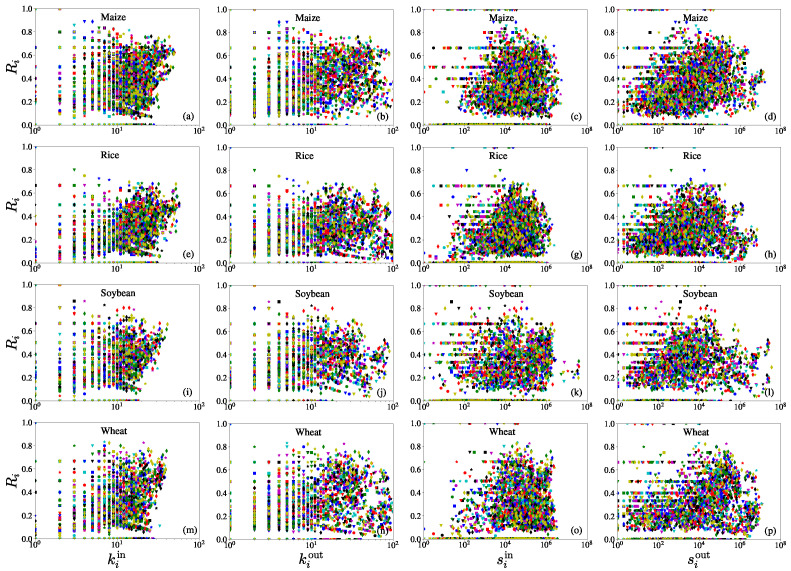
Scatter plots of the reciprocity coefficients of economies in the maize trade network with respect to the corresponding node attributes. (**a**,**e**,**i**,**m**) Reciprocity coefficient $R_{i}$ versus in-degree $k_{i}^{\mathrm{in}}$. (**b**,**f**,**j**,**n**) Reciprocity coefficient $R_{i}$ versus out-degree $k_{i}^{\mathrm{out}}$. (**c**,**g**,**k**,**o**) Reciprocity coefficient $R_{i}$ versus in-strength $s_{i}^{\mathrm{in}}$. (**m**,**h**,**l**,**p**) Reciprocity coefficient $R_{i}$ versus out-strength $s_{i}^{\mathrm{out}}$. Rows from top (**a**–**d**) to bottom (**m**–**p**) represent the international trade networks for maize, rice, soybean, and wheat. Different colors of the symbols correspond to different years. The in-degree and the reciprocity coefficient are positively correlated when the in-degree is large.

After calculating the reciprocity coefficient $R_{i}$ of economies in each iCTN, we focused on relationships between the reciprocity coefficients $R_{i}$ and the in-degree $k_{i}^{\mathrm{in}}$, the out-degree $k_{i}^{\mathrm{out}}$, the in-strength $s_{i}^{\mathrm{in}}$, and the out-strength $s_{i}^{\mathrm{out}}$. From Figure 8, we found that for each network, with the broadly distributed reciprocity coefficients (spanning from 0 to 1), the out-degree, the in-strength, and the out-strength do not have liner relationships with reciprocity coefficients. We also found that, as shown in Figure 8 (a, e, i, m), the in-degree and the reciprocity coefficient are positively correlated when the in-degree is large. This means that economies with more import trade partners usually have higher reciprocity coefficients.

### 3.6. Clustering Coefficient

The clustering coefficient is one of the most important statistical tools for understanding the structure of a network [37,38]. Several studies have proposed different definitions of the clustering coefficient [39,40]. For a node *i*, the clustering coefficient is the fraction between the number of observed triangles to all possible triangles in one network [41]:

(11) $$c_{i}=\frac{2T_{i}}{k_{i}\left(k_{i}-1\right)-2k_{i}^{R}},$$

where $T_{i}$ is the number of directed triangles through node *i*, $k_{i}$ is the total degree of node *i*, and $k_{i}^{R}$ is the reciprocal degree of node *i*.

**Figure 9:**
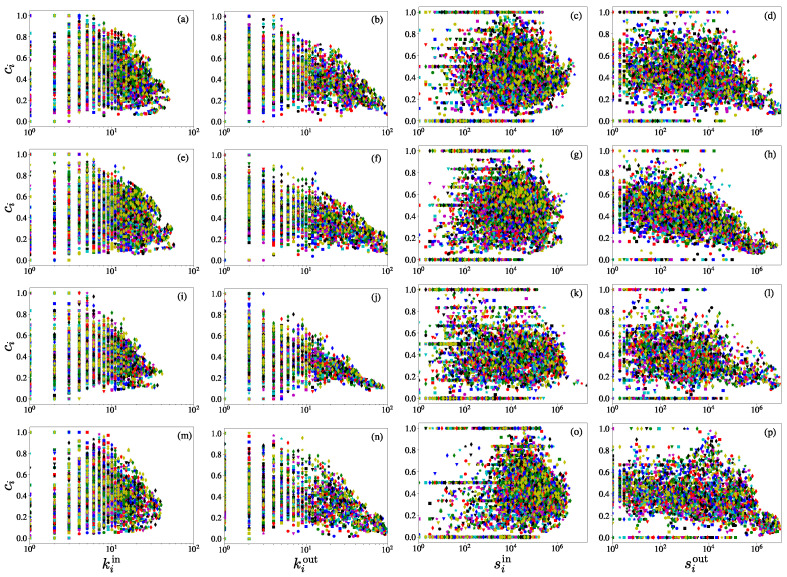
Scatter plots of the clustering coefficients of economies in the crop trade networks with respect to the corresponding node attributes. (**a**,**e**,**i**,**m**) Clustering coefficient $c_{i}$ versus in-degree $k_{i}^{\mathrm{in}}$. (**b**,**f**,**j**,**n**) Clustering coefficient $c_{i}$ versus out-degree $k_{i}^{\mathrm{out}}$. (**c**,**g**,**k**,**o**) Clustering coefficient $c_{i}$ versus in-strength $s_{i}^{\mathrm{in}}$. (**m**,**h**,**l**,**p**) Clustering coefficient $c_{i}$ versus out-strength $s_{i}^{\mathrm{out}}$. Rows from top (**a**–**d**) to bottom (**m**–**p**) represent the international trade networks for maize, rice, soybean, and wheat. Different colors of symbols correspond to different years. For each iCTN, the node clustering coefficient is negatively correlated with the in-degree and the out-degree. Furthermore, there are weak negative correlations between the clustering coefficient and the out-strength.

The node clustering coefficients, degrees, and strengths can all capture the micro-characteristics of the network. We analyzed the relationships between them and present the results in Figure 9. As shown in the first two columns, for each iCTN, the node clustering coefficient is negatively correlated with the in-degree and the out-degree. However, for some international trade networks, such as the total international trade network in 2000 [42] and the global transport network of crude oil [43], the node clustering coefficient exhibits no correlation with the total degree. As found by several other studies [44,45], economies that have more export partners (high out-degree) are less clustered than those having few partners. From the third column (c, g, k, o), the clustering coefficient was found to be uncorrelated with the in-strength. In contrast, there are weak negative correlations between the clustering coefficient and the out-strength. Therefore, the import and export trade values of an economy have different influences on its tendency to cluster [41]. It is also reported that the total strength of a node should be related to its clustering coefficient as a power law [46].

## 4. Conclusions

International food security has long been a global concern. Analyzing how iFTNs connect economies through import and export flows is an effective method for achieving food security [13]. We selected four major crops (maize, rice, soybean, and wheat) to construct the iCTNs, and used datasets from 1986 to 2018. For each crop, we constructed a network based on the international cross-border trade flows, where the nodes represent the economies participating in the international crop trade. We investigated the main microstructural properties of each crop network, including total trade value, node in- and out-degrees, node in- and out-strengths, link weight, reciprocity, and clustering coefficient. In the case of global crop trade (its values and trade partners), the directed weighted networks studied in this paper provide several crucial results and insights with respect to food security.

During the study period, the iCTNs became increasingly connected. It is obvious that there were more trade links in 2018 than before (Figure 1) and the structure of each network has significantly changed in the past three decades. More economies participated in the global crop trade and the trade values have dramatically increased over the past 33 years. In the world food trade networks [9,20] and the world trade networks [23,45], different iCTNs show different microstructural characteristics. This highlights the need to account for each crop trade network’s unique properties.

We investigated the distributions of node degrees and link weight, and found that each crop has a unique pattern of trade in each year. The degree distributions for each temporal iCTN do not always show power-law behavior, differing from the classical iFTN [42]. It was found that, for each iCTN, the total degrees *k* and the out-degrees $k^{\mathrm{out}}$ follow power-law distributions. However the in-degrees did not show significant power-law behavior. The link weights of yearly networks showed similar distributions, and followed power-law distributions in the maize, rice, and wheat trade networks. However, the distributions of the international soybean trade network did not have power-law behavior. It was interesting to explore the network of a single crop instead of total food and understand more details about the world food trade system.

By ranking node degrees each year, we found that economies which have the most trade partners are quite different among the crop trade networks. Some economies are always major participants in the trade networks, such as the United States, whereas some other economies fluctuate a lot in rankings. It can be seen that the United States has the highest rankings of node degrees in the four networks, especially in the maize, rice, and soybean trade networks (Figure 3). This means that the USA plays an important role in the international crop trade. After the collapse of the Soviet Union in 1991, Russia participated in international trade, and its number of trade partners gradually increased. It became the largest exporter of wheat worldwide in 2018. As for China, it is a large rice importer and it also imports soybeans and wheat from many economies. Overall, the rankings of node degrees in different iCTNs were distinct. In each network, the rankings of the total degree and the out-degree were steady, but the in-degree rankings fluctuated significantly.

Our findings about the national rankings of degrees are important, as they fundamentally reflect which economies occupy important positions in the crop trade system. It should help us reach a better understanding of potential vulnerabilities to some chaotic scenarios [9]. We could imagine a shock (such as extreme climate) to an economy that relies on its domestic rice production. What would the economy do to fill any gaps in domestic food supplies? The economy might resort to import partners to meet its food demands. Let us consider a more serious mess: that the largest rice exporter experiences major production loss. How would this scenario impact the international crop trade? Assuming that rice production in other areas does not increase, the global rice prices would increase without the release of rice reserves, and other alternative crops would also be affected [9].

Our analysis of the relationships among different topological properties has improved our understanding of the microstructural properties at play. We have shown that the in-degrees increase as the out-degrees increase, suggesting that economies expand overseas trade, including imports and exports, with the development of the international crop trade. The in-strength and the out-strength were almost uncorrelated in each iCTN. We can see that the in-strength was larger than the out-strength for most nodes, which indicates that most economies have a deficit in crop trade and need to import more than they export.

As noted in the above descriptions, some multi-year time characteristics of a specific crop network are different from those of the whole international trade network. To provide insights into food security under future shocks, it is better to analyze different crops rather than the whole food network.

## Data Availability

Publicly available datasets were analyzed in this study. This data can be found here: [http://www.fao.org] (accessed on 15 September 2021).
